# The use of specialty training to retain doctors in Malawi: A discrete choice experiment

**DOI:** 10.1016/j.socscimed.2016.09.034

**Published:** 2016-11

**Authors:** Kate L. Mandeville, Godwin Ulaya, Mylène Lagarde, Adamson S. Muula, Titha Dzowela, Kara Hanson

**Affiliations:** a Department of Global Health and Development, London School of Hygiene and Tropical Medicine, London, United Kingdom; b Johns Hopkins Project, Blantyre, Malawi; c Department of Public Health, School of Public Health and Family Medicine, College of Medicine, University of Malawi, Blantyre, Malawi; d Department of Clinical Services, Ministry of Health, Lilongwe, Malawi

**Keywords:** Malawi, Discrete choice experiment, Stated preferences, Human resources for health, Physician, Specialization

## Abstract

Emigration has contributed to a shortage of doctors in many sub-Saharan African countries. Specialty training is highly valued by doctors and a potential tool for retention. Yet not all types of training may be valued equally. In the first study to examine preferences for postgraduate training in depth, we carried out a discrete choice experiment as part of a cross-sectional survey of all Malawian doctors within seven years of graduation and not yet in specialty training. Over August 2012 to March 2013, 148 doctors took part out of 153 eligible in Malawi. Despite evidence that specialty training is highly sought after, Malawian junior doctors would not accept all types of training. Doctors preferred timely training outside of Malawi in core specialties (internal medicine, general surgery, paediatrics, obstetrics & gynaecology). Specialty preferences are particularly strong, with most junior doctors requiring nearly double their monthly salary to accept training all in Malawi and over six-fold to accept training in ophthalmology (representing a bundle of unpopular but priority specialties). In contrast, the location of work before training did not significantly influence most doctors' choices when guaranteed specialty training. Using a latent class model, we identified four subgroups of junior doctors with distinct preferences. Policy simulations showed that these preferences could be leveraged by policymakers to improve retention in exchange for guaranteed specialty training, however incentivising the uptake of training in priority specialties will only be effective in those with more flexible preferences. These results indicate that indiscriminate expansion of postgraduate training to slow emigration of doctors from sub-Saharan African countries may not be effective unless doctors' preferences are taken into account.

## Highlights

• Specialty training is valued by doctors and could be used to prevent emigration.
• Not all training, however, may be valued equally.
• Junior doctors preferred timely training outside Malawi in popular specialties.
• Latent class modelling identified four groups with distinct training preferences.
• Policy simulations show that these could be leveraged to improve retention.

## 1 Introduction

Of the 30 countries with the fewest doctors per population worldwide, 27 are in sub-Saharan Africa (World Health Organization, 2015). This paucity of doctors constrains the delivery of essential services and responses to new health threats, such as the rollout of antiretroviral treatment or the recent Ebola epidemic in West Africa (Sidibé and Campbell, 2015, World Health Organization, 2006). Factors contributing to the current situation include both low production and high emigration of doctors (Mullan et al., 2011, World Health Organization, 2006). Out of 105 medical schools surveyed in the Sub-Saharan African Medical Schools Study, half produced less than one hundred graduates in 2008 (Mullan et al., 2011). Of the doctors trained in sub-Saharan African medical schools, those who are now registered in the USA are equivalent to 22.7%, 26.2%, and 52.3% of the medical workforce in Ethiopia, Ghana, and Liberia respectively (Tankwanchi et al., 2013).

In response, there has been an unprecedented investment in undergraduate medical education in sub-Saharan Africa, with 58 new medical schools established since 1990 and many existing schools mandated to expand enrolment (Mullan et al., 2011). In contrast, there has been less focus on specialty training, the period of postgraduate training leading to accreditation as a specialist or general practitioner (World Federation for Medical Education, 2003, World Organization of Family Doctors, 2013). This is despite evidence that such training is highly valued by doctors and a strong driver for emigration (Willis-Shattuck et al., 2008). For example, a survey of 1619 non-European Union doctors working in the United Kingdom (UK) found that three out of four identified postgraduate training opportunities as their main reason for emigration (George et al., 2007). The desire to pursue specialty training increased the intention to emigrate within five years of qualification for medical students in eight low- and middle-income countries (LMIC), including five in sub-Saharan Africa (Silvestri et al., 2014). And nearly 90% of those doctors trained in sub-Saharan Africa but registered in the United States had completed their specialty training there rather than in their country of training (Tankwanchi et al., 2013). Yet only a third of sub-Saharan African medical schools offer such programmes, with a total of 1909 specialty training places for the 7861 graduates every year (Mullan et al., 2011). Offering more specialty training, therefore, is an attractive option for policymakers in sub-Saharan Africa seeking to maximise retention of their new medical graduates.

Retaining doctors is particularly important in Malawi, which has the second lowest ratio of doctors to people in the world (World Health Organization, 2015). Malawian doctors used to be trained in the UK, with the result that many never returned (Broadhead and Muula, 2002). In response, the one public medical school (College of Medicine-University of Malawi, COM) was established in 1991, with enrolment of first-year students reaching 105 in 2014 (Broadhead and Muula, 2002, University of Malawi, 2014)). Concerns over continued health worker emigration, however, led to the implementation of a six-year emergency programme in 2005 that included a 52% salary increase for doctors (Management Sciences for Health, 2010). Despite this, a study tracing COM graduates from 2006 to 2012 found that the odds of junior doctors being outside Malawi and the public sector increased with time after graduation, with most doctors outside Malawi in specialty training (Mandeville et al., 2014b). While enrolment to medical school tripled under the emergency programme, this was not matched by funding for specialty training (Management Sciences for Health, 2010). Given that previous studies have found that many medical students and junior doctors in Malawi intend to specialise, specialty training may prove a more effective tool for retention in the short-term than financial incentives (Bailey et al., 2012, Mandeville et al., 2012, Sawatsky et al., 2014). What is uncertain, however, is whether all kinds of specialty training would be equally effective. Depending on the availability of trainers for different specialties, Malawian doctors receive specialty training all in Malawi, all in South Africa (or other African countries), or split between the two (Zijlstra and Broadhead, 2007). Qualitative research has shown that junior doctors hold ambivalent views about specialty training undertaken entirely in Malawi (Bailey et al., 2012, Sawatsky et al., 2014). In addition, scholarships available in certain specialties - such as ophthalmology, anaesthetics or dermatology - have suffered from poor uptake from junior doctors. These specialties are a priority for training in terms of disease burden and available expertise in Malawi, yet are less established than the “*core*” specialties that dominate undergraduate training and internship: internal medicine, general surgery, paediatrics, and obstetrics & gynaecology (Bailey et al., 2012, Palmer et al., 2014, Schulze Schwering et al., 2014). Collectively, this evidence suggests that while junior doctors desire specialty training, not all training may be valued equally. Before specialty training can be used effectively to improve retention in Malawi, more information is needed on junior doctors' preferences towards different kinds of training. Discrete choice experiments are a quantitative method for eliciting preferences that are being used increasingly to inform health policy, particularly health workforce policy in low- and middle-income countries (Clark et al., 2014, de Bekker-Grob et al., 2012, Mandeville et al., 2014a, Ryan et al., 2008, Ryan et al., 2012). We therefore used a discrete choice experiment to investigate specialty training preferences of Malawian junior doctors.

## 2 Methods

### 2.1 Choice task design

To identify potential attributes and levels and obtain contextual information to inform the choice task design, we conducted a systematic review of DCEs focused on health workforce policy (Mandeville et al., 2014a) and a narrative review of literature investigating the health workforce crisis in Malawi. Possible attributes and levels were narrowed down through semi-structured interviews conducted with purposively sampling key informants (*n* = 18) and members of the target population (*n* = 19). Key informants were identified by snowball sampling and included policymakers, COM medical educationalists, specialist clinicians, hospital directors, and representatives of professional and hospital associations (Patton, 1990). To ensure common views were captured, junior doctors were sampled for maximum variation in age, job role, location and gender (Coast et al., 2004, Patton, 1990). We also interviewed doctors working outside the public sector (*n* = 4) and those that had left Malawi (*n* = 3) (Patton, 1990). Data were thematically analysed in NVivo version 10 (QSR International Pty Ltd, Doncaster).

Informed by this qualitative work, five attributes were selected that were both important to junior doctors and also potential policy levers. As our primary interest was the trade-off junior doctors would make between different aspects of specialty training, we used a generic rather than labelled design that presented two alternative hypothetical posts in the Malawian public sector (Job A versus Job B). In both jobs, a specialty training place would be guaranteed, but only after some time working in the public sector. This work would differ in the two job characteristics found to be most important to junior doctors and one policy lever:

• *Salary.* The monthly net salary paid before training. This included the basic salary for most junior doctors, MWK110,000 ($411 at exchange rates prevailing at the time, obtained from www.xe.com) as well as increments that were both realistic from a policy perspective and attractive to participants: 130,000 ($484); 160,000 ($596) or 200,000 ($746).
• *Job location.* The location of the hospital where this work would be undertaken. These were initially the two types of hospital in Malawi (central and district), but two subdivisions were made based on nuances emerging from the qualitative work. The final levels were major central hospital (located in the two main cities of Malawi, best facilities and most specialists), minor central hospital (located in smaller cities with poorer facilities and few specialists), district hospital near a major town (defined as less than 2 h' drive therefore providing proximity to urban amenities), and remote district hospital (more than 2 h' drive, limited amenities) (Bailey et al., 2012).
• *Time before training.* The amount of time required in the job before starting specialty training. While at least two years of training is nominally required before entry to specialty training, this has not always been enforced in the past. Some participants in the qualitative work were also prepared to work for up to five years in exchange for guaranteed specialty training. The final levels were therefore: 1, 2, 3 or 5 years. While best practice would be equally spaced levels, these were felt to be more realistic for participants.

The specialty training places would also differ in the two aspects found to be most important to junior doctors:

• *Training location.* Here, levels represented the most common options for Malawian doctors: training all in Malawi, split between Malawi and South Africa, all in South Africa, or all outside Africa.
• *Specialty.* Here, we wanted to investigate the trade-offs participants would be willing to make between different types of specialties and other aspects of a job. Employing named specialties as levels would provide little information other than their relative popularity. Instead, we formulated two levels as “1st choice” core specialty and “2nd choice” core specialty in order to explore the willingness of junior doctors in Malawi to compromise on future specialty. Only the core specialties were included here due to their familiarity to participants. So as to minimise misunderstandings around the terms, clear descriptions and instructions were included in the questionnaire. The third level was ophthalmology in order to investigate the incentives required to increase the uptake of less favoured but priority specialties. Finally, public health was included as a non-clinical specialty that is traditionally popular in Malawi, both due to the disease burden and a perception of access to well-paying jobs in non-governmental organisations. We had hoped to include family medicine to assess the attractiveness of generalist versus specialist training, however our target population were not sufficiently familiar with the specialty as it had only been recently introduced in Malawi.

**Fig. 1:**
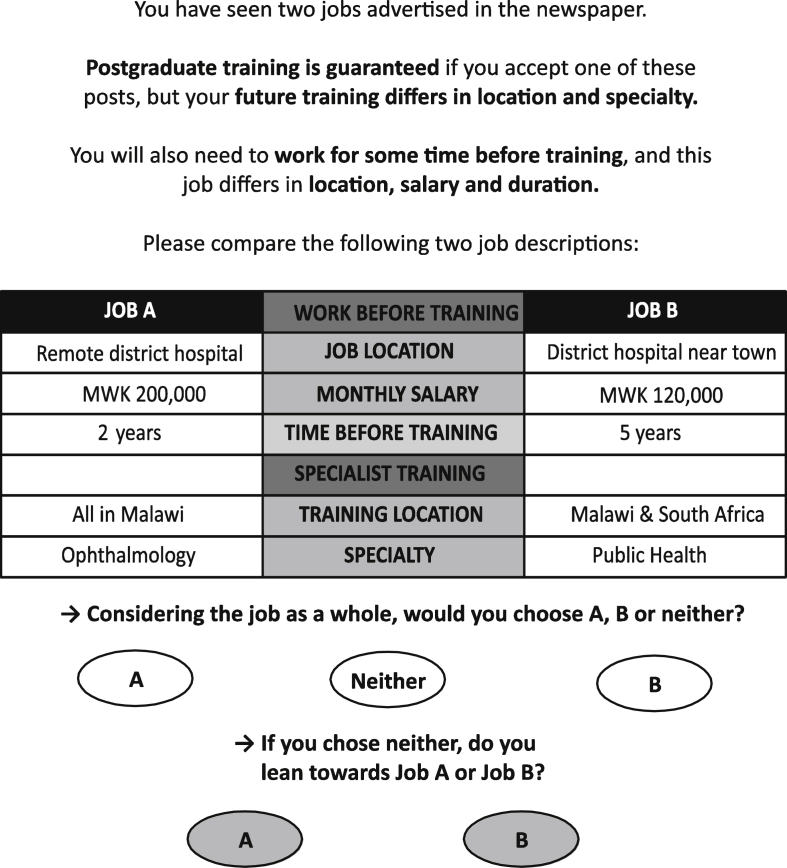
**An example choice task**.

We also included an opt-out option that participants could choose instead of making a choice between the job alternatives. The inclusion of an opt-out option avoids a “forced choice” that can overestimate the strength of preferences associated with alternatives (Carson et al., 1994, King et al., 2007, Louviere and Lancsar, 2009, Olsen and Swait, 1997, Ryan and Skatun, 2004). However, if the opt-out is chosen by the majority of participants, there is a risk of obtaining insufficient data. We therefore included a two-stage choice, first with an opt-out and then forcing participants to choose between the jobs on offer. Fig. 1 shows an example of the choice task.

### 2.2 Experimental design and piloting

Five attributes, all with four levels, gives 1024 possible combinations (4^5^) and 523,776 potential choice tasks ((1024 × 1023)/2) (M Ryan et al., 2012). With four levels, at least 16 choice tasks were needed for level balance. Our systematic review also identified that 16–20 choice tasks was the mode for previous DCEs administered to health workers (Mandeville et al., 2014a). We therefore used a D-efficient design in Ngene version 1.1.1 (ChoiceMetrics Pty Ltd, Sydney) to create a main effects design with 16 choice tasks. All parameter estimates were set to zero at this stage (see below). A multinomial logit model was specified, with the model averaging feature in Ngene used to evaluate designs for utility functions with and without an opt-out option.

In order to familiarise participants with the choice tasks and diminish the impact of learning effects on early responses (Lancsar and Louviere, 2006), participants completed ten practice exercises. These built up gradually from a choice between one attribute only to a full choice task. An accompanying questionnaire included questions on sociodemographic characteristics, current job and employment history, and attitudes towards specialist training. As part of the latter, we asked participants to indicate for 13 specialties whether: (i) they would want to train in it; (ii) they would consider training in it; or (iii) they would prefer not to train in it. Responses to these questions were used to construct a 13-point “specialty flexibility index”, with a positive response to (i) or (ii) scoring one point. Higher/lower scores on the index therefore indicate greater/lesser flexibility in specialty training choices.

We carried out a two-stage pilot: (i) pre-testing the questionnaire and choice tasks on 15 doctors who had participated in the qualitative work, with modification of wording and layout between participants based on feedback; (ii) a formal pilot of the initial DCE design on 16 doctors in order to obtain parameter priors (ChoiceMetrics Pty Ltd, 2012). Choice data from the pilot were analysed using a multinomial logit model in NLOGIT 4.0 (Econometric Software, Inc, Plainview). A design incorporating these parameter estimates with random distributions was run in Ngene using 1000 Sobol draws for 250,000 iterations in order to obtain the final survey design (Bliemer et al., 2008). To avoid primacy effects, two versions of choice tasks were produced with a reversed order and participants allocated to versions using a computer-generated random sequence.

### 2.3 Participants

The target population was all junior doctors (defined as within seven years of graduation) in Malawi who had not yet started specialty training. We had initially planned to use COM graduate lists along with Ministry of Health routine data to identify eligible participants, however these were quickly found to be out of date. We therefore complemented these routine data sources with up-to-date data on participants obtained through social media: an innovative process described elsewhere (Mandeville et al., 2014b). As our focus was on national retention policies, non-Malawian citizens and those who had completed any undergraduate training outside of Malawi were excluded (n = 20). Out of 279 graduates between 2006 and 2012, 153 were eligible (Mandeville et al., 2014b).

### 2.4 Data collection

Data collection was carried out over August 2012 to March 2013, with 2012 graduates interviewed after at least six months' work experience. Participants were invited to participate through social media, telephone or direct contact depending on their location. One of two research assistants administered the survey at participants' place of work. All participants received written and oral instructions, including an explanation of all attributes and levels. Participants completed paper survey booklets under the supervision of the research assistant. Participants received a small medical textbook as compensation for their time. Ethical approval was obtained from the research ethics committees of COM and the London School of Hygiene and Tropical Medicine, with informed consent obtained from all participants.

### 2.5 Analysis

Results were analysed using Stata 12 and NLOGIT 5.0 (Econometric Software, Inc, Plainview). Job location, specialty and training location were effects coded, with the estimate for the reference (omitted) level reconstructed by multiplying the values for all other levels by −1 and summing (Bech and Gyrd-Hansen, 2005). Salary and time before training were tested for linearity before coding continuously. While the effect for time before training was likely to be non-linear, model performance worsened considerably when coded as three separate parameters, therefore we chose continuous coding for model parsimony (Hensher et al., 2005). The opt-out option was coded with zero for all attributes.

An initial exploratory analysis was undertaken using a multinomial logit model (see supplementary file). This model does not take account of variation in preferences between respondents, however, for which mixed logit or latent class models are commonly used (Amaya-Amaya et al., 2008, de Bekker-Grob et al., 2012). Latent class models are semi-parametric models that assume that there are underlying subgroups (classes) of participants with similar preferences (Greene and Hensher, 2003). Membership of these classes characterised by unobserved (latent) variables, the nature of which may be inferred through observed variables. The analyst must stipulate the number of classes and which observed variables to include in the model. Within each class, choice probabilities are generated by the multinomial logit model (Greene, 2012). A posterior probability of belonging to each class is produced by the model for every participant. In order to describe each subgroup, it is possible to allocate each participant to a class based on their highest probability and then compare characteristics of participants across classes (Lagarde et al., 2014). In this way, latent class models are particularly well suited for policy-facing research, as distinct classes with specific policy recommendations creates a strong narrative for targeted policy actions.

We estimated latent class models with two to five classes, a panel specification, and observed variables that have been shown to be correlated with job and training preferences. These included: age, gender, year of graduation, marital status (married/relationship > 1 year vs. single/relationship < 1 year), children (yes vs. no), type of upbringing (rural vs. urban), current salary, number of dependents (includes extended family members in Malawi, <6 vs. ≥ 6), previous district work experience (yes vs. no), ever travelled outside of Africa (yes vs. no), parents' education level (either parent completed tertiary education vs. neither), time looking for a specialty training scholarship and the speciality flexibility index. A comparison of model fit measures (Akaike, corrected Akaike and Bayesian information criterions) showed that the best fitting model for the data comprised four latent classes and incorporated three observed variables: age, specialty flexibility index, and current salary (see Table 1).

We calculated marginal willingness to pay values, which can be interpreted as the compensating wage differential for a change in a single attribute change (Scott et al., 2013). Positive values indicate the amount of future income participants would give up in order to gain a unit increase or level change in an attribute, whereas negative values indicate the amount that a participant would want as compensation (Sivey et al., 2012). With effects coding, each coefficient represents the absolute utility of that level, rather than the marginal change from the reference level as with dummy coding. Therefore the marginal utility of a change between levels is given by the difference between their coefficients, which was then divided by the salary coefficient. For attribute k with four levels (l1 to l4), with level 1 as the omitted reference level, the willingness to pay for a change from level 1 to level 4 is then:

$$WTP_{k\gamma }=\frac{\beta_{kl4}-\left(-\beta_{kl2}-\beta_{kl3}-\beta_{kl4}\right)}{\beta_{\gamma }}$$

where *β*_*γ*_ is the marginal utility of income and (−*β*_*kl*2_ − *β*_*kl*3_ − *β*_*kl*4_) is the recovered coefficient for the omitted reference level (Scott et al., 2013). 95% confidence intervals were calculated using the delta method (Hole, 2007).

### 2.6 Policy scenarios

We then used simulation to predict the uptake of training posts under different hypothetical scenarios that could inform two key policy objectives in Malawi. The first was to maximise service in the public sector in exchange for training in favoured specialties. Here, we set the attributes of the first scenario to represent a common training pathway in Malawi: two years working in a district hospital near town at basic salary before training in a 1st choice core specialty in Malawi and South Africa. We then simulated the impact on job uptake of increasing lengths of mandatory service and remote location over four further scenarios. The predicted probabilities were simulated over two alternatives, Job A and the opt-out option, as there was no difference between Job A and Job B in this scenario.

The second examined strategies to increase the uptake of unpopular but priority specialties. Here, the attributes of Job A were fixed to represent the common training pathway above as the baseline scenario. Job B, in comparison, represents the type of training post in ophthalmology that had been reportedly difficult to fill (Scenario 1). Over Scenarios 2 to 4, Job B becomes increasingly incentivised through increased salary, shortened time before training, and training in Malawi and South Africa, with the uptake in each class noted each time.

## 3 Results

### 3.1 Participant characteristics

The response rate was 96.7% (149/153). There were very few graduates eligible from 2006 to 2007, as many were training or working outside Malawi (Mandeville et al., 2014b). As it was clear that we had defined our target population too widely for this choice problem, we excluded participants who had graduated in 2006 (8.3% of total graduates, 2/24) and 2007 (17.1%, 6/35). The final sample comprised 140 doctors, with 87 (62.1%) males and a median age of 25 years (SD 2.68). Gender data for the entire cohort shows 63.6% of these graduates are male, indicating that this sample is likely to be broadly representative (Mandeville et al., 2014b). The median monthly net salary was MWK 108,000 ($404), the starting salary in the public sector at that time. All doctors except one stated that they wished to specialise in the future, with three quarters currently looking for a training scholarship. When participants were asked which specialties they would consider training in, public health and epidemiology was the most popular specialty for training followed by the four core specialties, with psychiatry, dermatology and ophthalmology the least preferred (see supplementary file).

### 3.2 Preferences

All 140 participants completed all 16 choice tasks, giving 2240 observations. The opt-out option was used 831 times (37.1%). This is a substantial use of the opt-out option, however there was sufficient information to run the model with the opt-out option, therefore we did not analyse the forced choice data.

When the results of the four-class latent class model are examined (see supplementary file), doctors across all classes preferred higher salaries and less time before training. Training undertaken exclusively in Malawi is universally unattractive, with most preferring training in South Africa or outside Africa. The preferences for specialty are generally highly significant, indicating their importance in junior doctors' job choices, in comparison to job location that did not significantly influence doctors' choices for the most part. Most junior doctors would prefer to train in core specialties than ophthalmology, with divided preferences for training in public health.

**Fig. 2:**
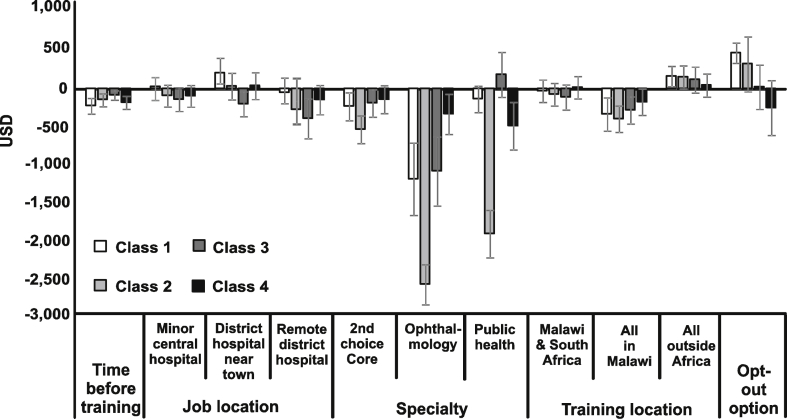
**Willingness to pay**. Positive values indicate the amount of future income participants would give up in order to gain a unit increase or level change in an attribute, whereas negative values indicate the amount that a participant would want as compensation. Reference levels = Major central hospital, 1st choice core specialty and training all in South Africa. 95% confidence intervals shown.

When willingness to pay values are compared across the four classes (Fig. 2), the strength of preferences for specialty in comparison to other attributes is striking. To train in ophthalmology rather than their first choice core specialty, junior doctors would need to be paid an extra MWK 89,589 to 691,804 ($334 to 2581) per month. To accept training in their second rather than first choice core specialty, doctors would require an extra MWK 37,470 to 62,573 ($140 to 538) per month. Training undertaken exclusively in Malawi would necessitate an extra MWK 90,257 to 106,760 ($282 to 398) per month for most doctors: a near doubling of basic salary. In contrast, doctors in class 1 would give up nearly half the basic salary (MWK 44,031, $164) to train outside Africa. For each year spent working before training, doctors would require an extra MWK 22,411 to 60,669 ($84 to 226) in monthly salary. Willingness to pay for changes in job location was smaller. Doctors in class 1 would give up MWK 56, 311 ($210) in order to work at a district hospital near a major town, whereas doctors in class 3 would require a near doubling in basic salary (MWK 105,841; $395) to work at a remote district hospital. Of note, the constant for the opt-out option was significant in class 1. This indicates that these doctors considered opting out regardless of the attributes on offer.

### 3.3 Subgroup descriptions

Based on posterior probabilities (of which 94% were between 0.9 and 1.0), we assigned each participant to a class and compared data obtained from the accompanying questionnaire across classes (Table 2). By comparing these with preferences obtained from the DCE (Fig. 2), broad descriptions of each subgroup can be arrived at in order to highlight key differences and policy implications. We describe the four subgroups seen here as the “rich rejecters”, “stubborn specialists”, “money motivated” and “pliant patriots”.

The “rich rejecters”, comprising Class 1 and a third of participants, were the only subgroup for whom the opt-out option was consistently more attractive than the jobs on offer and were also earning significantly more than their peers (1.5 times the sample mean salary). Although not significant, more doctors were outside the public sector in this group than the other three classes. These results suggest that public sector jobs, even with guaranteed specialty training and higher salaries, are unattractive to these doctors, who may be difficult to retain in the public sector in the long-term.

The “stubborn specialists”, Class 2 and also a third of participants, displayed the strongest specialty preferences of all subgroups and the highest disutility with accepting their 2nd rather than 1st choice core specialty. They also had strong preferences on the location of this training, requiring the highest compensation if all in Malawi. They tended to be younger than their peers and less likely to have worked at district level.

The “money motivated”, Class 3 and the smallest subgroup, had the largest preference for salary increases and would require the least compensation of all subgroups for more time working before training. Around a third of this subgroup had six or more dependents, significantly more than other subgroups. This was the only subgroup who preferred to train in public health rather than the other specialities, yet would also require the greatest compensation for working in a remote district hospital.

The “pliant patriots”, Class 4 and a fifth of the population, required the least compensation to train all in Malawi and were the only subgroup for which training all outside Africa or in ophthalmology did not significantly influence their choices. They also scored significantly higher on the specialty flexibility index and did not require significant compensation to train in their 2nd choice core specialty in contrast to the other subgroups. They tended to be older than their peers and had the lowest mean salary, but were no more likely to have family reasons to remain in Malawi.

### 3.4 Policy scenarios

**Fig. 3:**
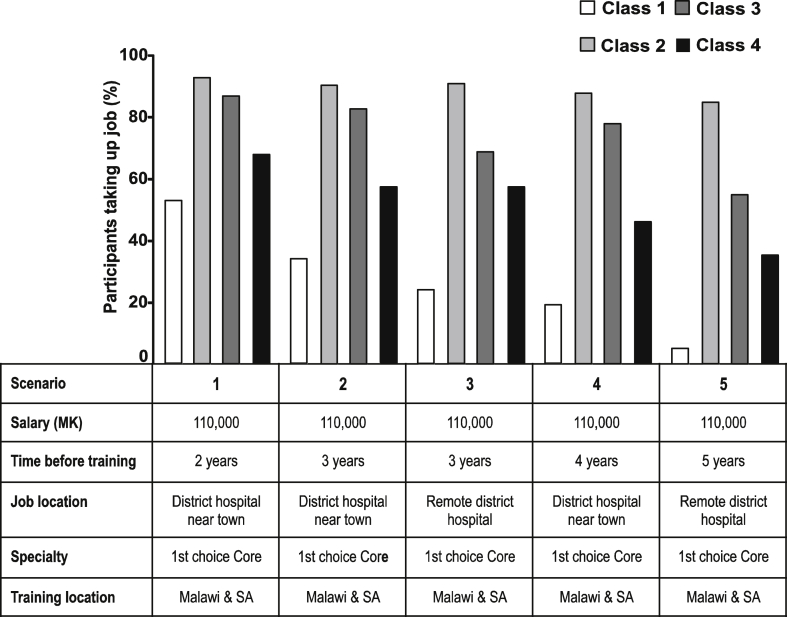
**Maximising public sector service in exchange for preferred training**. Scenario 1 represents a common training pathway in Malawi of two years working in a district hospital near town at basic salary before access to training in a preferred core specialty. Scenarios 2 to 5 simulate the impact of increasing lengths of mandatory service and remote location on job uptake. MWK = Malawian kwacha; SA = South Africa.

The first policy simulation assessed the extent to which service in the public sector could be maximised in exchange for training in popular specialties (Fig. 3). Our baseline scenario represents a common pathway to specialty training in Malawi with two years working in a district hospital at a starting salary, before training in a 1st choice core specialty in Malawi and South Africa. Nine out of ten stubborn specialists and money motivated doctors would accept this job, compared to just one in two rich rejecters. As the job evolves to one with longer mandatory service before training in less favourable locations, the uptake among rich rejecters drops steeply but remains considerable for the other three subgroups. Even facing five years in a remote district hospital at basic pay, more than a third of all junior doctors in these classes would accept this job in exchange for favoured training, rising to 85% among stubborn specialists.

**Fig. 4:**
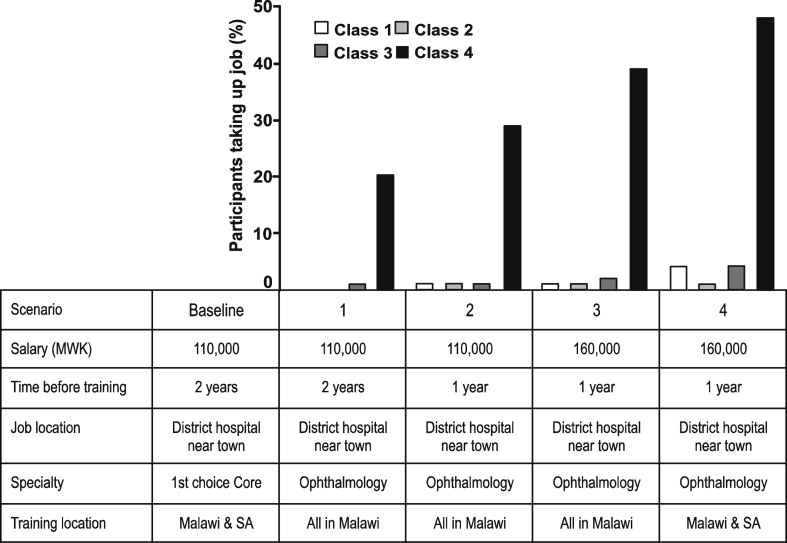
**Improving uptake of priority specialties**. The baseline scenario represents a common training pathway in Malawi. Scenario 1 represents the type of training post in ophthalmology that had been reportedly difficult to fill. Scenarios 2 to 4 offer increasing incentives to take up such a training post. MWK = Malawian kwacha; SA = South Africa.

The second series of policy simulations examined possible incentives to improve uptake of training in priority but unpopular specialties. Here, each job is compared against a baseline scenario to estimate the proportion of doctors that could be persuaded to take up training in ophthalmology over a common training pathway. As can be seen from Fig. 4, despite increasing incentives in terms of time before training, location of training, and salary, the uptake is minimal across all subgroups except the pliant patriots. In the final scenario, nearly one in two pliant patriots would choose a job training in ophthalmology with multiple incentives.

## 4 Discussion and conclusions

Junior doctors in Malawi do not value all specialty training equally, but prefer timely training outside of Malawi in core specialties. Specialty preferences are particularly strong, with most junior doctors requiring nearly double their monthly salary to accept training all in Malawi and over six-fold to accept training in ophthalmology compared to their preferred core specialty. In contrast, the location of work before training did not significantly influence most doctors' choice when guaranteed specialty training. We identified four subgroups of junior doctors with distinct preferences. Policy simulations showed that these can be leveraged by policymakers to maximise service in the public sector in exchange for guaranteed training in popular specialties, however incentivising the uptake of training in priority specialties will only be effective in those with more flexible preferences.

While previous discrete choice experiments have established the importance of postgraduate training to health workers, this is the first study to examine whether all postgraduate training is valued equally (Mandeville et al., 2014a). Our results indicate that indiscriminate expansion of postgraduate training to slow emigration of doctors from sub-Saharan African countries may not be effective unless doctors' preferences are taken into account. Considerable divergence in these preferences was revealed through the first use to our knowledge of a latent class model in this area of application. Interpretation of willingness to pay values, however, should be cautious given their sensitivity to the levels stipulated for the salary attribute (Ryan and Wordsworth, 2000, Sivey et al., 2012, Slothuus Skjoldborg and Gyrd-Hansen, 2003). Indeed, the level range could have been widened even further given the strong preferences for the type of specialty training, particularly among the stubborn specialists subgroup. A major limitation is the highly constrained sample size, which is likely to have limited the power of the model to identify significant effects of the attributes and observed variables. Moreover, a larger number of choice tasks divided into blocks would have been more appropriate to support model identification with the final number of eligible participants. This is a near complete census of the eligible population in Malawi, however, and in line with previous DCEs in LMIC (Mandeville et al., 2014a). Indeed, as finite population corrections are not possible for these models, it is likely that the significance levels shown here are conservative. Finally, the stated preferences elicited by discrete choice experiments may be seen to be inferior to the revealed preferences gleaned from controlled or observational studies. Yet controlled experiments, while aspirational, are politically difficult to perform on workforces. Observational studies are unable to distinguish the independent effects of attributes made possible through the experimental design of discrete choice experiments. Finally, in lower-income settings, resource constraints may restrict the options that can be offered to doctors, meaning that observable choices may not reflect true preferences.

Our findings concur with other studies demonstrating the value placed on postgraduate training by doctors and medical students compared to other job attributes, although these studies focused on attraction and retention in rural areas (Lagarde et al., 2013, Rao et al., 2013, Rockers et al., 2012, Vujicic et al., 2010, Vujicic et al., 2011). One other discrete choice experiment has explored specialty preferences, with Sivey et al. investigating the generic aspects of a specialty that increase its attractiveness to Australian junior doctors and finding a substantial effect of future earnings on specialty choice (Sivey et al., 2012). While these findings are from a high-income setting, the strong specialty preferences seen here may reflect perceived opportunities for private practice or, in the case of public health, well-paying jobs with non-government organisations (Bailey et al., 2012). Sivey et al. also found that doctors with higher levels of educational debt placed greater value on future earnings. Financial constraints may also have influenced the preferences of the money motivated subgroup, who supported more dependents, seldom opted out of the jobs on offer, and placed the highest value of all subgroups on public health training (despite disliking district-level work). While preferences for training location were not as strong as for the specialty itself, our results corroborate qualitative findings that most junior doctors in Malawi are reluctant to accept training all in Malawi (Bailey et al., 2012, Sawatsky et al., 2014). We did, however, identify a minority who were indifferent to training in higher-income settings and this proportion may increase as domestic training becomes more established. Three other studies have identified substantial heterogeneity in preferences for specialty training, although none using a latent class model (Lagarde et al., 2013, Rockers et al., 2012, Vujicic et al., 2010). In particular, Vujicic et al. examined job preferences of final-year medical students and graduated doctors (mean age 42) in Vietnam, finding that the most important attribute for students was guaranteed specialty training after five years, with a six-fold difference in willingness to pay compared to that of doctors (who most valued working in an urban area) (M Vujicic et al., 2010). We found that younger doctors had more fixed specialty preferences than older doctors, who were more willing to compromise on aspects of training. This suggests a critical window soon after graduation in which to leverage specialty training as an incentive, after which doctors place greater importance on other aspects of a job.

The broad conclusions from our results are likely to be generalisable to the many other sub-Saharan African countries at the same juncture in their domestic medical training (Mullan et al., 2011). For example, many countries in the region are struggling to increase their ophthalmologist workforce in line with agreed targets and a growing burden of eye disease (Palmer et al., 2014). Our results suggest that any investment into scaling up training should focus on those with more flexible preferences. These “pliant patriots” could be identified soon after graduation and targeted for fast-track training in priority specialties with an incentive package. In the same way, the firm training preferences of other junior doctors could be leveraged by stipulating several years of service in posts facing recruitment problems, e.g. remote district hospitals, as entry criteria for training in favoured specialties.

Our results provide clear evidence that postgraduate training is not a straightforward incentive, and future studies would benefit from more detailed exploration of this attribute. Future research following up career choices in this cohort would shed light on the predictive validity of subgroup preferences, e.g. whether pliant patriots tend to take up training in Malawi. It would also allow insight into the effectiveness of specialty training for retention in the long-term, for which there is currently no evidence to our knowledge. Finally, no choice is without cost, and the cost-effectiveness of providing specialty training in Malawi given the preferences identified here is also a key research question.

## Figures and Tables

**Table 1:** Comparison of model fit measures.

Number of latent classes	2	3	4	5
Parameters	28	44	60	76
Observations	2240	2240	2240	2240
Log-likelihood function	−2073.94	−1972.09	−1860.07	−1845.81
Pseudo R^2^	0.16	0.20	0.24	0.25
AIC	4203.90	4032.20	3840.10	3843.60
AICc	4204.63	4034.00	3843.46	3849.01
BIC	4363.88	4283.61	4182.99	4277.90

Notes: AIC = Akaike information criterion; AICc = Akaike information criterion with a correction for finite sample sizes; BIC = Bayesian information criterion.

**Table 2:** Characteristics of latent classes.

Characteristic	Class 1	Class 2	Class 3	Class 4
	N = 43 (30.7%)	N = 43 (30.8%)	N = 23 (16.0%)	N = 31 (22.6%)
*Sociodemographic characteristics*				
Female (N, %)	18 (41.9)	19 (44.2)	5 (21.7)	11 (35.5)
Median age in years (N, SD)**	25 (2.2)	24 (2.4)	25 (3.5)	26 (2.6)
Mean net monthly salary in MWK (N, SD)**^a^	147,193 (102,973)	123,884 (79,904)	112,827 (20,242)	108,633 (2822)
Working outside public sector (N, %)	4 (9.3)	1 (2.3)	2 (8.7)	1 (3.2)
District experience (N, %)**	12 (27.9)	4 (9.3)	9 (39.1)	10 (32.3)
Rural upbringing (N, %)	8 (18.6)	8 (18.6)	5 (21.7)	4 (12.9)
Married or relationship>1 year (N, %)	24 (55.8)	16 (37.2)	11 (47.8)	17 (54.8)
Children under 11 years old (N, %)	8 (18.6)	1 (2.3)	3 (13.0)	3 (9.7)
Six or more dependents (N, %)*	7 (16.3)	4 (9.3)	7 (30.4)	2 (6.5)
*Attitudes to specialty training*				
Specialty flexibility index (N, SD)***	6.0 (2.4)	6.0 (2.5)	5.0 (2.3)	7.4 (2.5)
Currently looking for specialty training funding (N, %)	32 (74.4)	33 (76.7)	15 (65.2)	21 (67.7)
Median months looking for funding (N, SD)^b^	3 (16.6)	3 (6.4)	3 (5.4)	4 (7.5)

Notes: N = number; SD = standard deviation; MWK = Malawian Kwacha; District experience signifies current or previous job at district level; One-way analysis of variance or chi-squared tests show significant differences across classes at the **5% or ***1% level; *Approaching significance with P-value of 0.056.

a Bartlett's test for unequal variance significant for original and log transformed data, therefore Kruskal-Wallis test used instead.

b Bartlett's test for unequal variance significant, therefore log transformation used instead.
