# Supplementary material for: The use of specialty training to retain doctors in Malawi: A discrete choice experiment
Source: Soc Sci Med. 2016 Nov;169:109–18. doi: 10.1016/j.socscimed.2016.09.034 (PMC5080456; doi:10.1016/j.socscimed.2016.09.034)
Supplement: Supplementary file 1 [file mmc1.pdf]

SUPPLEMENTARY INFORMATION

This document provides additional methodological details and results. The first section gives further details on the study population. The second section outlines the results used in the specialty flexibility index. The final section provides the multinomial logit and latent class model coefficient estimates.

1. Study population

Table A1 summarises participant characteristics and Figure A1 outlines the flow of participants.

Table A1 Participant characteristics

Characteristic	Observations	Number (%)	Mean (SD)	Range
Sociodemographic				
Male	140	87 (62.1)		
Age in years	140		25 (2.68)	21 - 36
Income in MWK	137		108,000 (74,465)	100,000 – 600,000
Position	140			
Intern		78 (55.7)		
Hospital medical officer		9 (13.6)		
District medical officer		20 (14.3)		
District health officer		11 (7.9)		
Outside public sector		12 (8.6)		
Attitudes to postgraduate training				
Desire to specialise	140	139 (99.3)		
Currently looking for scholarship	139	101 (72.7)		
Months looking for scholarship	100		8.9 (10.1)	1-50

Notes: *SD = standard deviation; MWK = Malawian Kwacha*

Figure A1 **Flow of participants through study**

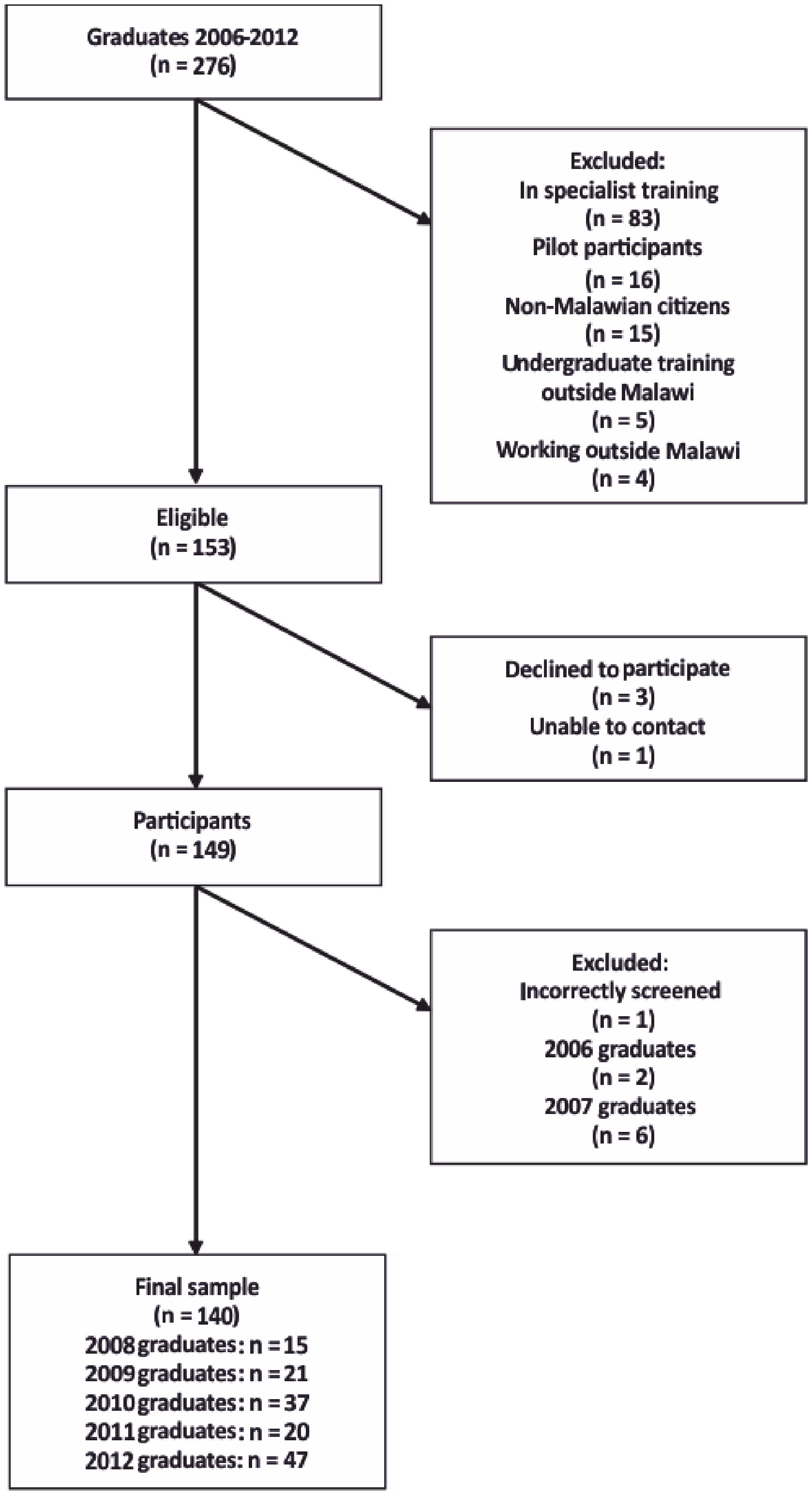

2. **Specialty preferences**

As part of the accompanying questionnaire, we asked participants to indicate for 13 specialties whether: (i) they would want to train in it; (ii) they would consider training in it; or (iii) they would prefer not to train in it. Figure A2 shows these specialty preferences. Responses to these questions were used to construct a 13-point "specialty flexibility index", with a positive response to (i) or (ii) scoring one point. Higher/lower scores on the index therefore indicate greater/lesser flexibility in specialty training choices.

Figure A2 Specialty preferences of participants

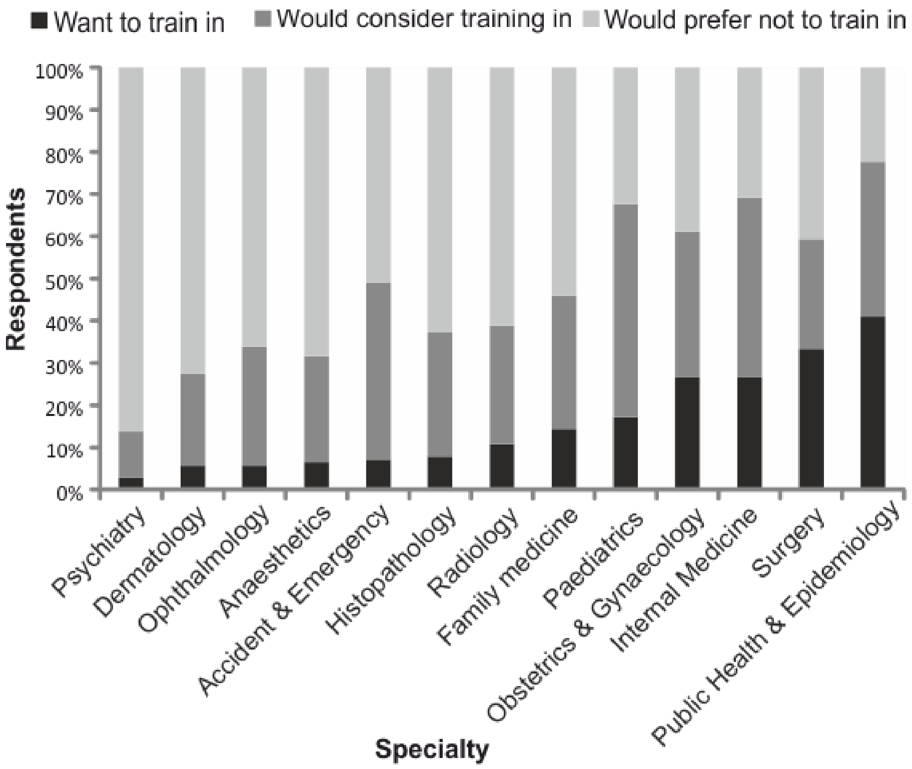

3. Econometric analysis

The full survey results were explored initially with a multinomial logit model. These results and willingness to pay values are shown in Table A2. All coefficients were of the expected sign. All are statistically significant at the 5% level or less, except for the parameters for minor central hospital and public health, indicating that these variables did not significantly influence participants' job choices in this model. Doctors preferred higher salaries, less time before training and working in a major central hospital or a district hospital near town. They preferred training outside of Malawi in their first- or second-choice core specialty, with training in ophthalmology bringing significant disutility.

We then went on to estimate latent class models, with the best fitting model containing four classes. The results of this model are shown in Table A3. The coefficients for the class membership variables indicate that, compared to the participant average, doctors in class 3 tended to be less flexible in their specialty preferences and doctors in class 2 tended to be younger (approaching significance at 0.053). The salary variable was not significant in any class, but its inclusion led to a better fit.

Table A2 Multinomial logit model results

**Number of observations**	2240		
**Number of parameters**	12		
**Log-likelihood function**	-2250.53		
**Pseudo R^2**	0.08		
**AICc**	3849.94		
**Bayesian information criterion**	4593.63		
**Attribute**	**Coefficient**	**SE**	**P-value**
Salary (MWK 10,000)	0.074**	0.009	0.000
Time before training (year)	-0.343*	0.029	0.000
Job location			
Major central hospital	0.079	[b]	[b]
Minor central hospital	-0.035	0.056	0.539
District hospital near town	0.163**	0.057	0.005
Remote district hospital	-0.207**	0.066	0.002
Training location			
All in South Africa	0.109	[b]	[b]
Malawi & South Africa	0.129*	0.058	0.025
All in Malawi	-0.539**	0.061	0.000
All outside Africa	0.230**	0.152	0.000
Specialty			
1st choice Core,[a]	1.025	[b]	[b]
2nd choice Core[a]	0.477**	0.054	0.000
Ophthalmology	-1.412**	0.090	0.000
Public health	-0.090	0.060	0.132
Opt-out option	0.337*	0.152	0.026

Notes: *AICc = Akaike information criteria with a correction for finite sample sizes; MWK = Malawian kwacha; SE = standard error; *Significant at 5% level;**Significant at 1% level or less; [a]See text for explanation; [b]Reference level, therefore only coefficient can be calculated from other parameter coefficients in category*

Table A3 Latent class model results

**Class**	**1**			**2**			**3**			**4**		
**Class probability**	0.307			0.308			0.160			0.226		
	**Coefficient**	**SE**	**P-value**	**Coefficient**	**SE**	**P-value**	**Coefficient**	**SE**	**P-value**	**Coefficient**	**SE**	**P-value**
**Job preferences**												
Salary (MK 10,000)	0.124**	0.027	0.000	0.070**	0.027	0.003	0.137**	0.035	0.000	0.094**	0.025	0.000
Time before training (year)	-0.752**	0.076	0.000	-0.277**	0.096	0.004	-0.306**	0.109	0.005	-0.457**	0.084	0.000
Job location												
Major central hospital	-0.209	[b]	[b]	0.157	[b]	[b]	0.674	[b]	[b]	0.125	[b]	[b]
Minor central hospital	-0.113	0.172	0.509	-0.0170	0.145	0.907	0.162	0.257	0.530	-0.114	0.129	0.377
District hospital near town	0.490**	0.181	0.007	0.217	0.153	0.156	-0.064	0.186	0.733	0.225	0.134	0.093
Remote district hospital	-0.168	0.188	0.371	-0.357	-0.357	0.062	-0.772*	0.319	0.015	-0.236	0.149	0.112
Training location												
All in South Africa	0.167	[b]	[b]	0.150	[b]	[b]	0.251	[b]	[b]	0.068	[b]	[b]
Malawi & South Africa	0.073	0.167	0.663	0.010	0.153	0.950	-0.155	0.202	0.444	0.113	0.119	0.344
All in Malawi	-0.952**	0.168	0.000	-0.598**	0.193	0.002	-0.783**	0.211	0.000	-0.367*	0.148	0.013
All outside Africa	0.712**	0.145	0.000	0.438**	0.156	0.005	0.687**	0.265	0.010	0.186	0.124	0.133
Specialty												
1st choice Core[,a]	1.296	[b]	[b]	2.364	[b]	[b]	0.999	[b]	[b]	0.617	[b]	[b]
2nd choice Core[a]	0.520**	0.174	0.003	1.353**	0.131	0.000	0.303	0.189	0.108	0.243	0.148	0.085
Ophthalmology	-2.661**	0.304	0.000	-2.485**	0.410	0.000	-2.984**	0.410	0.000	-0.235	0.201	0.242
Public health	0.845**	0.175	0.000	-1.232**	0.212	0.000	1.682**	0.302	0.000	-0.625**	0.157	0.000
Opt-out option	1.571**	0.445	0.000	0.611	0.464	0.187	0.082	0.562	0.884	-0.632	0.340	0.063
**Class membership variables**												
Age	-0.089	0.114	0.434	-0.318	0.164	0.053	0.186	0.156	0.232	-	-	-
Specialty flexibility index	-0.199	0.164	0.225	-0.213	0.191	0.264	-0.657*	0.288	0.023	-	-	-
Current salary (MK10,000)	0.156	0.130	0.231	0.142	0.146	0.332	-0.179	0.155	0.250	-	-	-

Notes: *MK = Malawian kwacha; *Significant at 5% level; **Significant at 1% level or less; [a]See main paper for explanation; [b]Reference level, therefore only coefficient can be calculated from other parameter coefficients in category*
